# Antimicrobial Resistance Profile and Genotypic Characteristics of *Streptococcus suis* Capsular Type 2 Isolated from Clinical Carrier Sows and Diseased Pigs in China

**DOI:** 10.1155/2015/284303

**Published:** 2015-05-03

**Authors:** Chunping Zhang, Zhongqiu Zhang, Li Song, Xuezheng Fan, Fang Wen, Shixin Xu, Yibao Ning

**Affiliations:** ^1^National Antimicrobial Resistance Monitoring Laboratory in Bacteria of Animal Origin, Department of Inspection Technology Research, China Institute of Veterinary Drug Control, Beijing 100081, China; ^2^China Animal Disease Control Center, Beijing 100125, China

## Abstract

*Streptococcus suis* serotype 2 is an important zoonotic pathogen. Antimicrobial resistance phenotypes and genotypic characterizations of *S. suis* 2 from carrier sows and diseased pigs remain largely unknown. In this study, 96 swine *S. suis* type 2, 62 from healthy sows and 34 from diseased pigs, were analyzed. High frequency of tetracycline resistance was observed, followed by sulfonamides. The lowest resistance of *S. suis* 2 for *β*-lactams supports their use as the primary antibiotics to treat the infection of serotype 2. In contrast, 35 of 37 *S. suis* 2 with MLS_B_ phenotypes were isolated from healthy sows, mostly encoded by the *ermB* and/or the *mefA* genes. Significantly lower frequency of *mrp*+/*epf*+/*sly*+ was observed among serotype 2 from healthy sows compared to those from diseased pigs. Furthermore, isolates from diseased pigs showed more homogeneously genetic patterns, with most of them clustered in pulsotypes A and E. The data indicate the genetic complexity of *S. suis* 2 between herds and a close linkage among isolates from healthy sows and diseased pigs. Moreover, many factors, such as extensive use of tetracycline or diffusion of Tn*916* with *tetM*, might have favored for the pathogenicity and widespread dissemination of *S. suis* serotype 2.

## 1. Introduction

*Streptococcus suis *is an important swine pathogen leading to big loss in pig production worldwide [1]. Among 35 serotypes currently identified, serotype 2 has gained more attention for the high prevalence and mortality rates in swine and human and is considered as an emerging zoonotic agent [2]. Furthermore, epidemiological surveillance has confirmed that* S. suis *type 2 can transmit from carrier pigs to humans [3, 4]. Clinically healthy carrier sows, harboring* S. suis* type 2, are considered as the major source of infection for their offspring [5]. Since the infection of* S. suis* type 2 commonly occurred among suckling and weaned piglets, it is essential to investigate the association of antimicrobial resistance profile and genotypic characteristicsof isolates from carrier sows and diseased pigs.

In the absence of effective vaccines to fight against* S. suis*, antimicrobial agents have become increasingly important in treating and controlling the infection of* S. suis* type 2. Of these, *β*-lactams, tetracyclines, sulphonamides, and macrolides are the most common antimicrobials used for the prevention and treatment of streptococcal infection in pig production. But inappropriate use of antibiotic has led to the development of resistance of* S. suis* to these drugs worldwide [6–9]. Furthermore, coresistance to tetracyclines and macrolides/lincosamides in human* S. suis* isolates was observed, and the relevance of Tn*916*-like conjugative transposon in coresistant mechanisms and clone diffusion have been studied [9, 10]. However, to date, few reports about coresistance to these three classes of antibiotics and the Tn*916 *family were found in swine* S. suis *serotype 2.

In recent years, research on* S. suis* type 2 has mainly concentrated on its potential virulence factors and pathogenic mechanisms. Many factors, including polysaccharide capsule (*cps*), muramidase-released protein (*mrp*), the extracellular protein factor (*epf*), the suilysin (*sly*), glyceraldehyde-3-phosphate dehydrogenase (*gdh*), and a fibronectin/fibrinogen-binding protein (*fbp*), were found to be associated with virulence of* S. suis* 2 [11–14]. Of these,* mrp*,* epf*, and* sly* were considered as the most relevant factors to the pathogenesis of* S. suis* by many researchers [15–17]. Recently, other new putative virulence factors, such as surface-associated subtilisin-like serine protease (*SspA*), factor H-binding protein (*fhb*), have also been identified [18, 19]. However, our knowledge about pathogenesis of serotype 2 is still limited despite the increasing number of studies.

To deeply understand* S. suis* type 2 infection, molecular typing methods are usually applied to identify individual isolates and establish genotypic characterization. Currently, many typing methods, such as randomly amplified polymorphic DNA (RAPD) [20], ribotyping [21], multilocus sequences typing (MLST) [22, 23], and genome sequencing [24], have been used to define the diversity of* S. suis* and to distinguish virulent from nonvirulent isolates. Of these, pulsed-field gel electrophoresis (PFGE) is one of the most powerful molecular typing methods. Many researches had been done to compare differences between* S. suis *from different animals, and genetic diversity was found on the basis of isolates of the different pathogenic serotypes [25–27]. However, a thorough characterization of serotype 2 isolates from healthy sows and diseased pigs has not thus far been reported.

The aim of the study is to investigate antimicrobial resistance phenotypes, genotypes, and genetic patterns of* S. suis* type 2 from clinical carrier sows and diseased pigs. Furthermore, the phenotypic and genotypic characterizations of these isolates were compared. To the best of our knowledge, this is the first integrative report about resistance profile and genetic diversity of* S. suis* serotype 2 from clinically healthy sows and diseased pigs.

## 2. Materials and Methods

### 2.1. *S. suis* Type 2 Isolates

A total of 96 swine* S. suis* serotype 2 were included in this study. 62 isolates were recovered from tonsils of clinically healthy sows of 15 epidemiologically unrelated farms in 10 regions (Jiangsu, Sichuan, Guangdong, Guangxi, Anhui, Henan, Hebei, Jiangxi, Shandong, and Beijing) from March 2005 to November 2012. Of the 34* S. suis* serotype 2 obtained from diseased pigs, 31 outbreak isolates were from 6 epidemiologically unrelated farms in Sichuan, Jiangsu, Anhui, Henan, and Beijing during 2005–2008, and three historical isolates (C55604, C55609, and C55612) were provided by China Veterinary Culture Collection Center (CVCC). All isolates were determined as* S. suis *types 2 by biochemical characteristics (API 20 strep, bioMérieux SA, France) and sera agglutination reaction (special antisera provided by the Statens Serum Institut, Copenhagen, Denmark) and further confirmed as serotype 2 by positive PCR for the genes coding for the 16S rRNA of* S. suis* and for the capsule of* S. suis* serotype 2 (*cps2J*) [23].

### 2.2. Antimicrobial Susceptibility

Antimicrobial susceptibility testing was performed using the standard broth microdilution method [28]. The following antimicrobial agents, the representatives of commonly used drug classes in China, were tested (with dilution ranges in parentheses): penicillin G (0.06–8 *µ*g/mL), ampicillin (0.12–16 *µ*g/mL), ceftiofur sodium (0.12–16 *µ*g/mL), enrofloxacin (0.015–4 *µ*g/mL), clindamycin (0.03–4 *µ*g/mL), erythromycin (0.06–8 *µ*g/mL), tilmicosin (0.5–64 *µ*g/mL), chloramphenicol (1–128 *µ*g/mL), tiamulin fumarate (0.25–32 *µ*g/mL), tetracycline (0.06–8 *µ*g/mL), sulfisoxazole (16–512 *µ*g/mL), and trimethoprim/sulfamethoxazole (0.5/9.5–16/304 *µ*g/mL).* Streptococcus pneumoniae *ATCC 49619 was used as the quality control strain. The isolate was defined as an MLS_B_-phenotype that was found to be resistant to erythromycin, lincomycin, and tilmicosin.

### 2.3. Genotyping

PCR virulence genotyping of all isolates was performed for the* mrp*,* epf*,* sly*,* orf2*,* fbp,* and* gdh* genes. Moreover, 85 tetracycline-resistant (tet^r^) isolates were also analyzed for the presence of tetracycline-resistant genes (*tetK*,* tetL*,* tetM, *and* tetO*), macrolide-resistant genes (*ermB, ermA*, and* mefA*), lincosamide-resistant gene (*lnuB*), and Tn*916*-like transposon family (*intTn* and* xis*), respectively. Positive and negative controls were included with each PCR assay. Target genes and the corresponding primer sequences were listed in Table 1. Confirmation of the amplicons was determined by DNA sequencing and the results were analyzed using BLAST software (http://www.ncbi.nlm.nih.gov).

### 2.4. PFGE Analysis

All* S. suis* serotype 2 isolates were typed using pulsed-field gel electrophoresis (PFGE) as described previously [31, 32] with minor modifications. Briefly, cell culture was suspended in cell suspension buffer (100 mM Tris : 100 mM EDTA, pH8.0) to 10 McFarland standards. Proteinase K was then added at final concentration of 0.5 mg/mL and mixed with equal volume of molten 1% Seakem Gold Agarose before loading into the plug mold. After solidification, the agarose plugs were submerged in cell lysis buffer (CLB, 50 mM Tris : 500 mM EDTA, pH8.0 + 1% sarkosyl) with lysozyme (1 mg/mL) before incubation at 37°C for 12 h and then CLB with proteinase K (0.5 mg/mL) was added and cultured at 54°C for 2 h with vigorous shaking. After washing, the plugs were sliced and then digested in fresh restriction buffer with the enzyme* Sma*I (50 U/*µ*L) at 25°C for 12 h. The electrophoresis was performed with CHEF-DR III system (Bio-Rad) at 14°C under the electric field strength 6 v/cm for 19 h with pulse time ramping from 2.2 s to 63.8 s.

*Salmonella enterica *serovar Braenderup H9812 restricted with* Xba*I was used for molecular weight and size determinations. Similarities between restriction endonuclease digestion profiles were analyzed using BioNumerics software (Applied Maths, Kortrijk, Belgium) with Dice coefficients and clustering by an unweighted paired group with arithmetic averaging. The dendrogram of PFGE patterns of isolates tested was drawn with a 1.5% position tolerance and 1% optimization. And the cluster cutoff was set at an 85% similarity level. The different PFGE fingerprints were assigned as different uppercase letters.

### 2.5. Statistical Analysis

SPSS for Windows, version 16.0, was used for statistical analysis. The frequencies of antibiotic resistance, resistant genes, and virulence-associated factors were compared between the isolates from healthy carrier sows and from diseased pigs. The chi-square (or Pearson chi-square) and Fisher exact tests were used when appropriate. Differences were considered significant when two-sided *P* value was less than 0.05.

## 3. Results

### 3.1. Antimicrobial Susceptibility Testing and Detection of Resistance Genes

The collections of 96* S. suis* 2 were tested for susceptibility to 12 antimicrobials (Table 2). High frequency of resistance was observed for tetracycline, followed by sulfonamides. 57 and 46 isolates from healthy sows and 28 and 18 from diseased pigs were resistant to tetracycline and sulfisoxazole, respectively. These two antimicrobial agents had MIC_50_ values (>8, 512 *µ*g/mL, resp.) equal to or higher than the highest concentration tested. The lowest resistant rates of* S. suis* 2 for *β*-lactams were found, and all isolates were susceptible to ampicillin. Data also suggested the high incidence rates of resistance for macrolides and lincosamides in the isolates from healthy carrier sows. Among 85 tet^r^ isolates, 37 had MLS_B_ resistance phenotypes, 35 from carrier sows and the remaining two from diseased pigs. No inducible resistance pattern was discovered. A Significantly higher occurrence of MLS_B_ resistance was observed in* S. suis* 2 from carrier sows than those from diseased pigs (*P* < 0.005).

Antimicrobial resistance patterns and resistant determinants for tetracyclines, macrolides/lincosamides were analyzed in Table 3. No detection of* tetK* and* tetL* genes was observed among tet^r^ *S. suis* 2. The* tetM* gene was found among 77 and the* tetO* gene among 51 of 85 tet^r^ *S. suis* type 2. None of erythromycin- and clindamycin-resistant isolates carried* ermA *or* lnuB*. 35* S. suis* 2 with MLS_B_-phenotype were shown to be* ermB *and* tetM* positive and 18* mefA* positive. Neither* ermB* nor* mefA* was detected in the non-MLS_B_  tet^r^ isolates. Presence of the* ermB* gene was strongly associated with MLS_B_-phenotype of* S. suis* 2. Significant carrier difference of the* tetO* gene, but not* tetM*, was observed between erythromycin-resistant (32/37) and erythromycin-susceptible (19/48) isolates.

None of 85 tet^r^ *S. suis* serotype 2 was positive for the* xis *gene. 38 tet^r^ isolates (26 from diseased pigs and the remaining 12 from healthy sows) carried the* intTn* gene. Unexpectedly, all isolates (38/96) with* intTn* gene were resistant to tetracycline only, while they were susceptible to macrolides/lincosamides. And higher frequency of* intTn* with* tetM* was observed in isolates from diseased pigs (22/34) compared to those from healthy carrier sows (12/62).

### 3.2. Virulence-Associated Genes Analysis

96 serotype 2 isolates were cloned and screened for the presence of the* mrp*,* epf*,* sly*,* orf2*,* fbp,* and* gdh* genes. The distribution of virulence-associated genes was reported in Table 3. All isolates were positive for the virulence genes coding for* fbp* and* orf2* and negative for* gdh*. 41, 58, and 47 of 62 healthy sows isolates harbored* mrp, epf*, and* sly*, respectively. In contrast, the* mrp, epf*, and* sly* genes were detected in all* S. suis* 2 recovered from diseased pigs but two* epf*-negative isolates.

Six kinds of virulence genotypes were obtained in* S. suis* capsular type 2 from carrier sows, with high frequency of* mrp+/epf+/sly+* (30/62)*, mrp−*/*epf*+*/sly*+ (15/62), and* mrp*+/*epf*+*/sly*− (9/62) (Table 4). All* S. suis* type 2 from diseased pigs had the virulence genotype of* mrp+/epf+/sly+* with two exceptions of* mrp*+/*epf−/sly*+. Significantly lower carrier rate of* mrp*+/*epf*+/*sly*+ genotype was observed among isolates from healthy sows compared to those from diseased pigs (*P* < 0.005).

### 3.3. PFGE Typing

**Figure 1:**
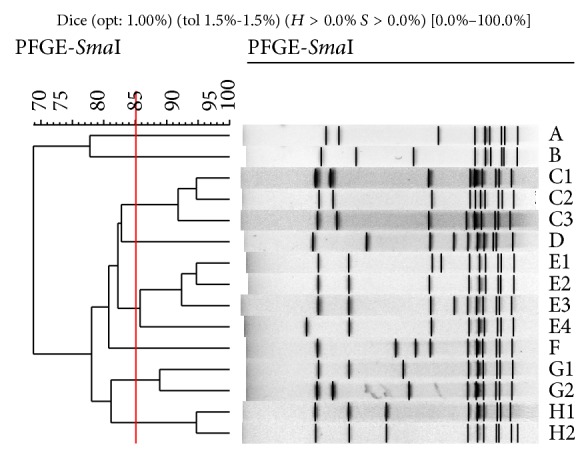
Genetic relationship of representative bands of* S. suis* type 2 isolated from clinically healthy sows and diseased pigs. Dendrogram showed the genetic relatedness of* S. suis* type 2 pulsotypes A to H2. The cluster cutoff (red line) was set at an 85% similarity level. Numbers at the upper left indicate percent similarity.

On the basis of an investigation of 96* S.  suis* type 2, PFGE typing produced 15 different fingerprints, which were grouped into types A to H (Figure 1). Pulsotypes C1–C3, E1–E4, G1-G2, and H1-H2 were considered to be respectively related, with more than 85% similarity.

In contrast, significant difference was observed between the isolates from diseased pigs and healthy sows. All isolates from diseased pigs were classified as pulsotypes A, E, and G (Tables 3 and 5). Pulsotypes A and E predominated in diseased pigs and were detected in 32 of 34* S. suis* 2 isolates. For isolates from clinically healthy sows, pulsotype G (21/64) was the most frequently observed one, followed by types C (13/64), H (10/64), D (8/64), E (4/64), F (4/64), and B (2/64). Interestingly, the same pulsotype, E or G, was found in* S. suis* 2 both from healthy sows and from diseased pigs. For example, isolates C55604 (from historically diseased pigs) and one isolate (farm GD-2, from healthy sows) with 100% homogeneity were present in pulsotype E2 (Table 5).

All* S. suis* serotype 2 from the same farm, including those isolated in different years, shared the pulsotype with more than 85% similarity; most of them had the identical PFGE patterns. 6 isolates from farm GD-1 in 2006 and 2009 were assigned to PFGE type C, and the similar results were observed for pulsotypes D, G2, H1, and E3. Complex relationship among* S. suis* 2 isolates from different farms was discovered. Three isolates from Beijing had the pulsotype (E4) different from Sichuan and Jiangsu isolates (pulsotype A) even if these* S. suis* serotype 2 were isolated during the largest outbreak of human* S. suis* 2 infection occurring in 2005. However, some pulsotypes were more frequently isolated and exhibited a wide distribution over herds compared to others. For instance, 13 isolates from different farms in Henan, Jiangsu, and Shandong provinces were classified as PFGE subtype G1, and similar results were obtained for patterns C1, C2, and D. Furthermore,* S. suis *serotype 2 with types G1, C1, C2, and D were resistant to tetracycline and positive for the* tetM* gene, although four different virulence genotypes,* mrp*+/*epf*+/*sly*+,* mrp*−/*epf*+/*sly*+,* mrp*+/*epf*−/*sly*+, and* mrp*−/*epf*+/*sly*−, were involved (not shown in tables).

## 4. Discussion

### 4.1. Resistant Phenotypes and Genotypes of* S. suis* 2

The lowest resistance of* S. suis* serotype 2 for *β*-lactams was in accordance with other discoveries [7, 8], supporting their use as the primary drugs to treat the infection of swine* S. suis* serotype 2. The resistance to tetracyclines in* S. suis* has become a major worldwide problem, closely related to the widespread use of tetracycline in swine production. And tetracycline-resistance has been considered to be an important cofactor in the selection of resistance to macrolides/lincosamides [9]. In this study, 85 of the 96 isolates were resistant to tetracycline, 37 of which were coresistant to macrolides and lincosamides antibiotics. This indicated less frequent coresistance of* S. suis *2 to tetracyclines and macrolides/lincosamides. And the similar results were also observed by other researchers [6, 10, 33]. Furthermore, 35 of 37* S. suis* 2 with MLS_B_-phenotype were isolated from healthy sows, indicating the presence of selective pressure of antimicrobial agents since tilmicosin and tylosin were widely used as swine feed additives in China. In contrast, most of tet^r^ isolates from diseased pigs in backyard without feed additives were susceptible to macrolides/lincosamides.

Tetracyclines resistance in streptococci is mediated by ribosomal protection proteins or efflux proteins, encoded mainly by the* tet* genes [34]. Neither* tetK* nor* tetL* was detected in this study, which was consistent with other recent analyses [10]. The* tetM *and* tetO* genes, both coding for ribosomal protection protein, were widespread in tet^r^ *S. suis*, and higher carrier rate of the* tetM* gene than the* tetO* gene was also observed in other studies [9, 35].

Resistance to macrolide of streptococcal clinical isolates is commonly encoded by ribosomal methylase (*erm*) genes and efflux (*mef)* genes [29]. The* ermB* gene was found in all but two erythromycin-resistant isolates, confirming its frequency in* S. suis* type 2 in China [9]. High number of MLS_B_-resistant isolates with the* ermB* gene is in agreement with other research findings [30, 36]. To our knowledge, no large scale survey concerning the distribution of the* mefA* gene in* S. suis* type 2 has been described in the literature. Wierzbowski et al. found that the* mefA* gene conferred low-level resistance of* Streptococcus pneumoniae* to macrolides only (M phenotype) [36]. In this study, about half of* S. suis* 2 with MLS_B_ resistance harbored the* mefA* gene. In the view of the fact that all* mefA*-positive isolates showed MLS_B_-phenotype and harbored the* ermB* gene,* mef*-mediated resistance can be obscured by the effects of the* erm* gene in phenotypic tests.

### 4.2. Tn*916*-Like Transposon Family

Tn*916 *is one of the most extensively studied conjugative transposons in gram-positive bacteria. The integrase* intTn* gene is responsible for transposition, and the excisase* xis* gene may increase the frequency of excision but is not required [37]. In this present study, it is interesting that the* intTn* gene was detected only in erythromycin- and clindamycin-susceptible isolates and no* xis* genes were detected, indicating the absence of relatedness between presence of Tn*916*-like conjugative transposon and macrolides/lincosamides resistance phenotypes of* S. suis* type 2.

It is worth noting that the* tet *genes are often carried by Tn*916*-like conjugative transposon and erythromycin resistance genes are also carried on the same element, which contributes to the coresistance of streptococci to tetracyclines and macrolides. Previous studies have investigated the association between* tetM*/*tetO* and* ermB*/*mefA* in* S. pneumoniae *or* S. pyogenes*, and conjugal transfer experiments demonstrated that* tetM*/*tetO *and* ermB*/*mefA* were consistently cotransferred by Tn*916*-Tn*1545*-like transposons [38, 39]. However, the* intTn *gene was not detected among 35 MLS_B_ isolates with* tetM* and* ermB* in this study, and therefore, elements other than the Tn*916* family might be associated with coexistence of these two genes in* S. suis* serotype 2 with MLS_B_ phenotype.

The* tetM* and* intTn *genes, the markers for the Tn*916* family of elements [40], were harbored by 22 of 36 isolates from diseased pigs in the study. Ye et al. also found that* tetM* was associated with Tn*916* in* S. suis* type 2 from human outbreak [9]. Thus, the presence in* S. suis *2 of elements related to Tn*916* with* tetM* could play an important role in the pathogenicity of this bacterial pathogen. Further studies are necessary to monitor the spread of these elements in* S. suis* serotype 2 circulating in environments.

### 4.3. Virulence-Associated Factors

To further characterize the molecular features of the isolates from the diseased pigs and healthy sows, six virulence-associated genes were detected. The* gdh* gene was not detected in any isolates, revealing that presence of this gene may be not necessary for these isolates included in this study. Different results from other studies indicated that carriage of the* gdh* gene was associated with multilocus sequence type (ST) or origins of isolates [41]. Moreover, the* fbp* gene was present in all* S. suis* type 2, and similar results were also observed by de Greeff et al. [12], who suggested that the* fbp* gene is present among most serotypes except for serotypes 32 and 34. Thus, virulence difference between the isolates from clinically healthy sows and those from diseased pigs may lie in the frequency of the* mrp*,* epf*, and* sly* genes, since all* S. suis* serotype 2 analyzed in the present study also carried the* orf2* gene. Interestingly, more than 60% isolates from clinically healthy sows harbored the* mrp*,* epf*, and* sly* genes. The higher carrier rate of virulent genotype of* mrp*+/*epf*+/*sly*+ in* S. suis* type 2 from diseased pigs compared to healthy sows showed that these three genes together may contribute to differentiating the virulence of serotype 2, which is in accordance with results of other epidemiological reports [9, 17]. However, since 30 of 62 isolates from clinical carrier sows were also genotyped* mrp+/epf+/sly+ *in this study and similar result from healthy pigs was obtained by other researchers [15], it is necessary to perform further studies to specify the virulence of serotype 2* mrp+/epf+/sly+* isolates.

### 4.4. PFGE Subtyping

Many studies showed that PFGE could effectively detect relationship between genetic background, virulence traits, and epidemiologic implication of many bacterial pathogens [27, 31]. In this study, all isolates were typed by PFGE and clustered in 8 pulsotypes.* S. suis* serotype 2 from diseased pigs showed more homogeneously genetic patterns than those from healthy sows, with most of them clustering in pulsotypes A and E. In contrast, the majority of isolates from healthy sows clustered in the patterns G, C, and H, presenting a high level of divergence.

Despite the genetic diversity observed, four PFGE profiles, G, E, A, and C (accounted for 72 of 96 isolates), were more frequently observed than other patterns. These four prevalent PFGE profiles were isolated from diseased pigs and healthy sows from 9 provinces in different years (Table 5), indicating their widespread distribution in Chinese swine population. Of these, PFGE pattern G1 was unique and predominant among* S. suis* serotype 2 isolates from four epidemiologically unrelated herds, suggesting the existence of a prevalent clone. In consideration of the characteristic of their* tetM*-positive tetracycline resistance, diffusion has probably provided considerable advantages by the use of antimicrobial agents in different farms or horizontal acquiring of genetic elements, such as Tn*916 *with* tetM* [9, 42]. In addition,* S. suis* serotype 2 with pulsotype G1 showed dissimilarity at virulence genotypes, including* mrp+/epf+/sly*+,* mrp+/epf−/sly*+, and* mrp−/epf+/sly*+, which demonstrates that factors other than antimicrobial susceptibility might have favored for its diffusion.

The identical PFGE pattern (C3, D, G2, H1, or E3) was detected for the isolates from the same farm in different years (Table 5), and the persistent dissemination of* S. suis* serotype 2 clone within the herd of swine could be confirmed [26]. Furthermore, pulsotype E1 (E2 or G1) was found among the isolates from historical diseased pigs and from healthy sows. In our opinion, this can be explained with two main reasons. Firstly, after a long-term adaption to the healthy sows,* S. suis* 2 gradually lost virulence and finally became avirulent. Secondly, as a kind of conditional pathogenic bacteria, the possibility that healthy carrier sows harbor* S. suis* 2 is capable of causing disease under specific circumstances cannot be ruled out [5], suggesting a close linkage of* S. suis* serotype 2 from healthy sows and diseased pigs.

Taken together, the present study is the first systematic description of resistant phenotypes and genetic genotypes of* S. suis* type 2 isolated from clinically healthy sows and diseased pigs in China. Significant differences of MLS_B_ resistance phenotype, virulence-associated genotypes, and PFGE pulsotypes were observed between isolates from clinical carrier sows and those from diseased pigs. The results indicate that *β*-lactams are still the primary drugs to treat the infection of swine* S. suis* serotype 2. The unique and predominant PFGE types within and between herds show persistent dissemination of* S. suis* 2 and a close linkage among isolates from healthy sows and diseased pigs. Moreover, our data also support the contention that extensive use of tetracycline and horizontal acquiring of genetic element, Tn*916* with* tetM*, could act as a selective factor for the pathogenicity and widespread diffusion of serotype 2 [9, 42].

## Figures and Tables

**Table 1:** Target genes and PCR primers used in this study.

Gene target(s)	Primer sequence (5′-3′)	Amplicon size (bp)	Reference
Macrolide/Lincosamide resistance genes			
*ermB *	GAAAAGGTACTCAACCAAATA	639	[29]
	AGTAACGGTACTTAAATTGTTTAC		
*ermA *	GAAGTTTAGCTTTCCTAA	395	[29]
	GCTTCAGCACCTGTCTTAATTGAT		
*mefA *	AGTATCATTAATCACTAGTGC	346	[29]
	TTCTTCTGGTACTAAAAGTGG		
*lnuB *	CCTACCTATTGTTTGTGGAA	944	[29]
	ATAACGTTACTCTCCTATTC		

Tetracycline resistance genes			
*tetK *	TATTTTGGCTTTGTATTCTTTCAT	1159	[26]
	GCTATACCTGTTCCCTCTGATAA		
*tetL *	ATAAATTGTTTCGGGTCGGTAAT	1077	[26]
	AACCAGCCAACTAATGACAATGAT		
*tetO *	AACTTAGGCATTCTGGCTCAC	519	[26]
	TCCCACTGTTCCATATCGTCA		
*tetM *	GAACTCGAACAAGAGGAAAGC	740	[26]
	ATGGAAGCCCAGAAAGGAT		

*IntTn* and *xis* genes			
*intTn *	GGTCTTCGTATTTCAGAGTTTGG	473	[30]
	GTTGCATGTGCGTAATAGTTCAG		
*xis *	AAGCAGACTGACATTCCTA	193	[30]
	GCGTCCAATGTATCTATAA		

Virulence-associated factors			
*mrp *	ATTGCTCCACAAGAGGATGG	188	[15]
	TGAGCTTTACCTGAAGCGGT		
*epf *	CGCAGACAACGAAAGATTGA	744	[15]
	AAGAATGTCTTTGGCGATGG		
*sly *	GCTTGACTTACGAGCCACAA	248	[15]
	CCGCGCAATACTGATAAGC		
*fbp *	GACGGATCCTTTTTACATCACATGACGG	247	this study
	CCGTCGACGTATTTCCGCAGAATCAT		
*orf2 *	CAAGTGTATGTGGATGGG	860	this study
	ATCCAGTTGACACGTGCA		
*gdh *	GGCGCCGAATTCGTCGACATTTAGCAATTTTTGCG	1039	this study
	CGCCGCGGATCCGTAGTTAAAGTTGGTATTAAC		

**Table 2:** Antimicrobial resistance profile of *S. suis* serotype 2 from clinically healthy carrier sows and diseased pigs.

Antimicrobials	MIC breakpoint^*^ (*µ*g/mL)			Healthy carrier sows (*n* = 62)		Diseased pigs (*n* = 34)		Total (*n* = 96)	
	S	I	R	MIC range	*n* ^∗∗^	MIC range	*n* ^∗∗^	MIC range	*n* ^∗∗^
Penicillin	0.12	0.25–2	4	≤0.06–>8	2	≤0.06–1	0	≤0.06–>8	2
Ampicillin	0.25	0.5–4	8	≤0.12–2	0	≤0.12–0.5	0	≤0.12–2	0
Erythromycin	0.25	0.5	1	0.12–>8	35	≤0.06–4	2	≤0.06–>8	37
Clindamycin	0.5	1-2	4	0.06–>4	35	0.06–>4	2	0.06–>4	37
Enrofloxacin	0.25	0.5–1	2	0.12–>4	6	0.06–1	0	0.06–>4	6
Tetracycline	2	4	8	0.5–>8	57	0.25–>8	28	0.25–>8	85
Ceftiofur	2	4	8	≤0.12–>16	3	≤0.12–2	0	≤0.12–>16	3
Tiamulin	16	—	32	≤0.25–>32	7	≤0.25–16	0	≤0.25–>32	7
Tilmicosin	16	—	32	1–>64	35	≤0.5–64	2	≤0.5–>64	37
Chloramphenicol	4	8	16	≤1–32	3	≤1–8	0	≤1–32	3
Sulfisoxazole	256	—	512	32–>512	46	32–>512	18	32–>512	64
Trimethoprim/ sulfamethoxazole	2/38	—	4/76	≤0.5/9.5–>16/304	13	≤0.5/9.5–16/304	2	≤0.5/9.5–>16/304	15

^∗^MIC breakpoints were taken from Clinical and Laboratory Standards Institute standards (CLSI).

S: susceptible; I: intermediate; R: resistant.

^**^Number of resistance isolates.

**Table 3:** Distribution of antimicrobial resistance patterns, resistant determinants, virulence factors, and pulsotypes of *S. Suis* type 2 isolates.

Origin of isolates	Resistant pattern^a^	Number of isolates	Number of isolates with resistant determinants^b^					Number of isolates with virulence genes^c^ (%)			PFGE subtypes (number of isolates)
			*intTn *	*erm*B	*mef*A	*tet*M	*tet*O	*mrp *	*epf *	*sly *	
Healthy sows (*n* = 62)	Ery^r^Til^r^Cli^r^Tet^r^	35	0	33	16	33	30	16	33	26	C (5), D (8), F (4), G (10), H (8)
	Ery^s^Til^s^Cli^s^Tet^r^	22	12	0	0	20	12	20	20	17	B (2), C (8), G (8), E (4)
	Ery^s^Til^s^Cli^s^Tet^s^	5	/	/	/	/	/	5	5	4	G (3), H (2)
	Total	**62**	**12**	**33**	**16**	**53**	**42**	**41**	**58**	**47**	B (2), C (13), D (8), E (4), F (4), G (21), H (10)

Diseased pigs (*n* = 34)	Ery^r^Til^r^Cli^r^Tet^r^	2	0	2	2	2	2	2	2	2	A (2)
	Ery^s^Til^s^Cli^s^Tet^r^	26	26	0	0	22	7	26	24	26	A (10), E (14), G (2)
	Ery^s^Til^s^Cli^s^Tet^s^	6	/	/	/	/	/	6	6	6	A (2), E (4)
	Total	**34**	**26**	**2**	**2**	**24**	**9**	**34**	**32**	**34**	A (14), E (18), G (2)

^a^Ery^r^: erythromycin resistant; Til^r^: tilmicosin resistant; Cli^r^: clindamycin resistant; Tet^r^: tetracycline resistant; Ery^s^: erythromycin susceptible; Til^s^: tilmicosin susceptible; Cli^s^: clindamycin susceptible; Tet^s^: tetracycline susceptible.

^b^The *ermA*, *lnuB*, *tetK*, *tetL*, and *xis* genes were not detected in the isolates included in this study.

^c^All isolates carried the *fbp* and *orf*2 genes and none harbored the *gdh* gene.

/: Not detected.

**Table 4:** Virulence genotypes of 96 *S. suis* capsular type 2.

Virulence genotypes^*^	Number of *S. suis* capsular type 2		
	Healthy sows	Diseased pigs	Total
*mrp*+/*epf*+/*sly*+	30	32	62
*mrp*+/*epf*−/*sly*+	2	2	4
*mrp*−/*epf*+/*sly*+	15	0	15
*mrp*−/*epf*+/*sly*−	4	0	4
*mrp*+/*epf*+/*sly*−	9	0	9
*mrp*−/*epf*−/*sly*−	2	0	2

Total	62	34	96

^∗^All isolates had the genotype of fbp+/orf2+/gdh−.

**Table 5:** PFGE patterns of 96 *S. suis* serotype 2 isolates included in this study.

Origin	PFGE pattern (*n* ^#^)	Number of isolates	Farm number^##^	Isolated years
Healthy sows	B (2)	2	AH-2	2011
			
	C1 (4)	2	GD-1	2009
		2	GX-1	2007
			
	C2 (5)	3	SD-1	2006
		2	HB-1	2007
			
	C3 (4)	4	GD-1	2006, 2009
			
	D (8)	2	BJ-2	2008
		6	HN-3	2009, 2010
			
	E1 (3)	3	GD-2	2009
			
	E2 (1)	1	GD-2	2007
			
	F (4)	4	HB-2	2007
			
	G1 (13)	7	HN-1	2006
		1	JS-2	2009
		5	SD-2	2011
			
	G2 (8)	8	GX-2	2010, 2012
			
	H1 (8)	8	JX-1	2007, 2009
			
	H2 (2)	2	SC-3	2007

Diseased pigs	A (14)	8	SC-1	2005
		2	SC-2	2005
		4	JS-1	2005
			
	E1 (2)	2	C55609, C55612	/
			
	E2 (1)	1	C55604	/
			
	E3 (12)	12	AH-1	2007, 2008
			
	E4 (3)	3	BJ-1	2005
			
	G1 (2)	2	HN-2	2006

^#^Number of isolates with the same PFGE pattern.

^##^Farm number was named as capital letters (abbreviation of the province/region)—serial number. AH: Anhui; GD: Guangdong; GX: Guangxi; SD: Shandong; HB: Hebei; BJ: Beijing; HN: Henan; SC: Sichuan; JS: Jiangsu; JX: Jiangxi. C55604, C55609, and C55612 were provided by CVCC.

/: isolated time was not provided.
